# Regulatory RNAs controlling vascular (dys)function by affecting TGF-ß family signalling

**DOI:** 10.17179/excli2015-423

**Published:** 2015-07-10

**Authors:** Kondababu Kurakula, Marie-Jose Goumans, Peter ten Dijke

**Affiliations:** 1 Department of Molecular Cell Biology, Cancer Genomics Centre Netherlands, Leiden University Medical Center, Leiden, The Netherlands

**Keywords:** microRNA, cardiovascular disease, transforming growth factor-ß, bone morphogenetic protein, endothelial cells, smooth muscle cells

## Abstract

Cardiovascular disease (CVD) is a leading cause of morbidity and mortality worldwide. Over the last few years, microRNAs (miRNAs) have emerged as master regulators of gene expression in cardiovascular biology and disease. miRNAs are small endogenous non-coding RNAs that usually bind to 3′ untranslated region (UTR) of their target mRNAs and inhibit mRNA stability or translation of their target genes. miRNAs play a dynamic role in the pathophysiology of many CVDs through their effects on target mRNAs in vascular cells. Recently, numerous miRNAs have been implicated in the regulation of the transforming growth factor-β (TGF-β)/bone morphogenetic protein (BMP) signalling pathway which plays crucial roles in diverse biological processes, and is involved in pathogenesis of many diseases including CVD. This review gives an overview of current literature on the role of miRNAs targeting TGF-β/BMP signalling in vascular cells, including endothelial cells and smooth muscle cells. We also provide insight into how this miRNA-mediated regulation of TGF-β/BMP signalling might be used to harness CVD.

## Introduction

Cardiovascular diseases (CVD) remains a predominant cause of morbidity and mortality worldwide in spite of our advances in the understanding of the etiology (Cohen et al., 2000[17]). Indeed, CVD are the number one cause of death throughout the world killing 17.5 million people in 2012 (WHO, 2012[88]). Although mortality rates related to CVD are significantly reduced due to the identification of major risk factors such as obesity, dyslipidaemia, diabetes, and hypertension, the prevalence of heart disease is greatly expanding (NHLBI, 2012[59]). This impending burden of the disease is stressing the need for further insight into the molecular mechanisms that contribute to the pathology of CVD as well as the search for innovative therapeutic agents for CVD prevention and treatment. 

microRNAs (miRNAs) are endogenous, well conserved, small noncoding RNA molecules (20-25 nucleotides) that regulate the expression of approximately 60 % of the mammalian genes at post-transcriptional level (Lewis et al., 2005[52]; Friedman et al., 2009[29]). It is well established that miRNAs bind to conserved regions in the 3′ untranslated region (UTR) of their target mRNAs and inhibit either mRNA stability or the translation of their target genes in a tissue- and development-specific manner. Each miRNA can target multiple mRNAs and numerous miRNAs can regulate one target mRNA, demonstrating the huge combinatorial complexity and regulatory potential of miRNAs (Bartel, 2009[6]; Dombkowski et al., 2011[25]). Both basic and clinical studies have demonstrated that miRNAs are involved in a diversity of biological processes such as apoptosis, proliferation, migration, and differentiation (van Rooij, 2011[83]). miRNAs are expressed in a tissue-, time- and context-dependent manner and can there by regulate many pathophysiological processes. Recent studies show that miRNAs are highly expressed in vascular cells and play crucial role in the development and progression of CVD. Understanding the effect miRNAs have on key transcription factors or genes that are associated with CVD will be pivotal for the development of innovative therapeutic strategies to treat CVD patients. 

Members of transforming growth factor β (TGF-β) family play key roles in many diseases, including CVDs such as hypertension, atherosclerosis, arteriovenous malformations (AVMs), and aneurysms (ten Dijke and Arthur, 2007[80]; Pardali et al., 2010[66]; Doetschman et al., 2012[24]). TGF-β family members have been implicated in controlling vascular morphogenesis and function. In vitro as well as in vivo studies have shown their important role in controlling vascular cells, including endothelial and mural cell proliferation, migration, and apoptosis (Heldin et al., 1997[38]; Heldin and Moustakas, 2012[39]). Like in other biological processes, TGF-β family members exert their effects on vascular cells in a time and context-dependent manner (Pardali and ten Dijke, 2012[67]; Akhurst, 2012[2]). Recent reports demonstrated that TGF-β signalling plays critical role in the regulation of several miRNAs. Moreover, numerous miRNAs also control expression of several members of the TGF-β family.

In this review, we summarize the recent advances on miRNAs that control TGF-β signalling in (cardio)vascular diseases. We only discuss briefly about the regulation of miRNAs by TGF-β, as this is covered by excellent recent reviews (Ha and Kim, 2014[35]; Hata and Liebermann, 2015[37]). We focus on the miRNAs that regulate TGF-β signalling in vascular cells, including endothelial cells (ECs) and smooth muscle cells (SMCs). We centre our review on two vascular diseases that are caused by perturbation in TGF-β family signalling, i.e. hereditary haemorrhagic telangiectasia (HHT) and pulmonary arterial hypertension (PAH), two haploinsufficiency diseases of which patients can have inactivating mutations in one allele of a TGF-β family (co)receptor. Inhibition of miRNAs that target these receptors may restore their expression to normal levels, which may be of therapeutic benefit. 

## TGF-β signalling

TGF-β signalling is involved in a vast majority of cellular processes and plays crucial role throughout life starting from gastrulation and body axis asymmetry to organ-specific morphogenesis and adult tissue homeostasis. The human TGF-β superfamily comprises 33 evolutionarily conserved pleiotropic cytokines including three TGF-β isoforms, activins, growth and differentiation factors (GDFs), and bone morphogenetic proteins (BMPs). TGF-β family members regulate many key cellular processes such as cell division, differentiation, migration, adhesion, organization and death, extracellular matrix (ECM) production, tissue homeostasis and embryogenesis in a context- and cell type-dependent manner. Disruption of TGF-β signalling has been implicated in several developmental disorders and diseases, including cancer, fibrosis, auto-immune and CVDs (Heldin et al., 1997[38]; Heldin and Moustakas, 2012[39]; Shi and Massagué, 2003[75]; Schmierer and Hill, 2007[72]; Goumans et al., 2009[31]; Derynck and Miazono, 2008[20]). 

All TGF-β ligands are secreted in an inactive form, but become biologically active when carboxy- terminal mature domain is released from interacting with the amino-terminal pro-domain. The biologically active TGF-β ligand is a dimer (ten Dijke et al., 2007[80]; Chang et al., 2002[15]). TGF-β family members regulate multiple cell signalling pathways by binding to a complex of two type II and two type I serine/threonine kinase transmembrane receptors and signals through both Smad-dependent and Smad-independent pathways. For example, different factors regulate the TGF-β/Smad signalling pathway in multiple steps. In mammals, seven type I receptors, also known as activin receptor-like kinases (ALKs), and five type II receptors have been reported (Heldin et al., 1997[38]; Shi and Massagué, 2003[75]; Schmierer and Hill, 2007[72]). Besides the two signalling receptors, also two co-receptors, betaglycan and endoglin, have been identified that present ligands to the type I and type II receptors (Goumans et al., 2009[31]; Wong et al., 2000[90]).

**Figure 1:**
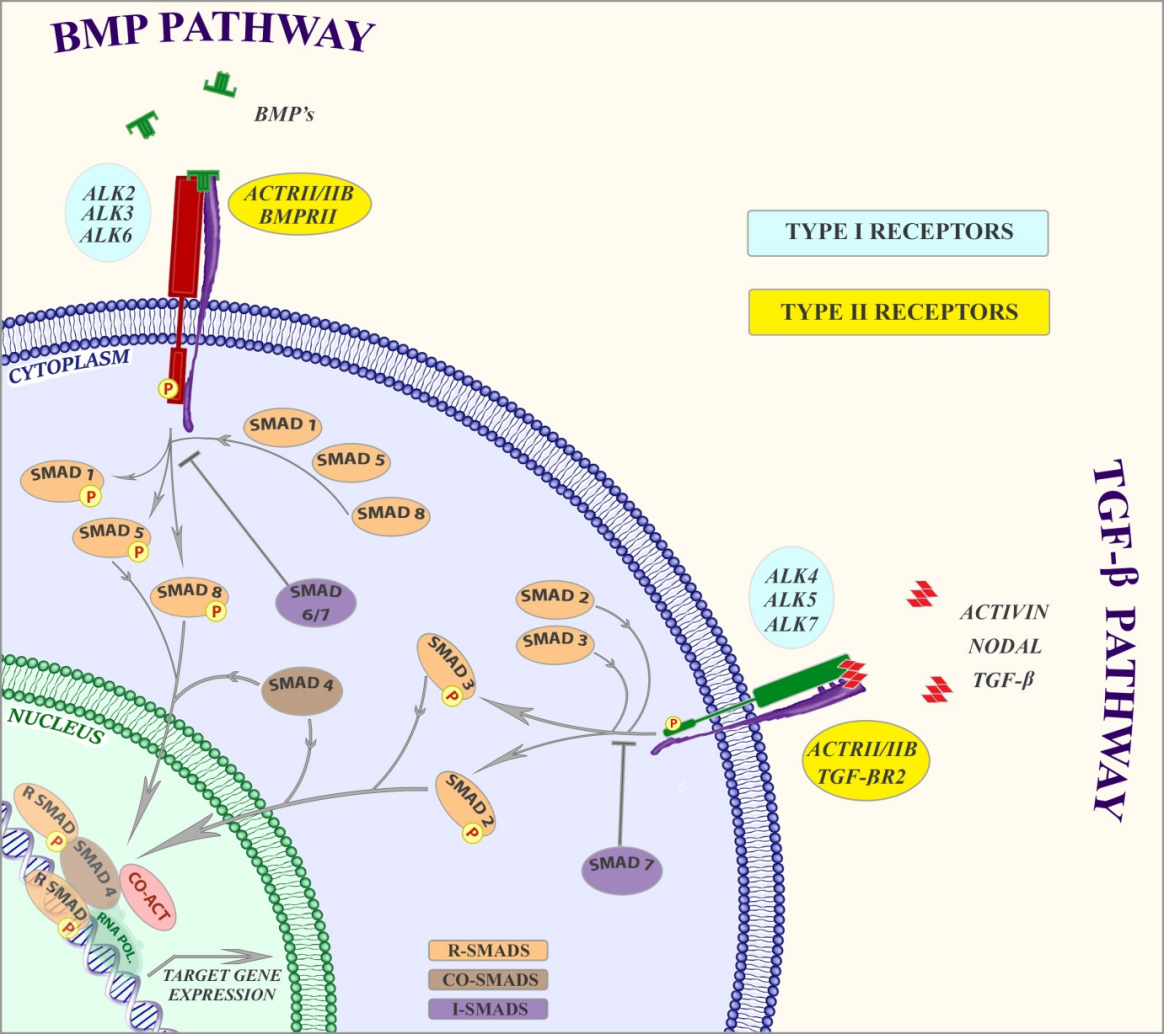
TGF-β superfamily signalling TGF-ß, activin or nodal ligands binds to a type II receptor dimer, which then recruit a type I receptor dimer. The type II receptor phosphorylates serine residues of the type I receptors leading to activation of the protein. Activated type I receptors phosphorylate its downstream targets SMAD2/3 (i.e. R-Smads) which then form hetero-oligomeric complexes with the co-SMAD, SMAD4 and translocate to the nucleus to bind DNA at sequence-specific DNA motifs to regulate gene expression. The heteromeric SMAD complex also interacts with various co-regulators for transcriptional activation or repression. SMAD7 can inhibit the phosphorylation of SMAD2 and SMAD3. BMP signalling functions in a similar pattern. BMP6 and BMP7 bind to their type II receptor which then recruits the type I receptors, ALK3 or ALK6. In contrast, BMP2 and BMP4 bind first to their type I receptor which then recruits the type II receptor ACTRII or BMPRII. Activation of the receptor complex results in phosphorylation of the receptors and then leads to phosphorylation of SMAD1/5/8 which form hetero-oligomeric complexes with the SMAD4. The heteromeric SMAD complex translocate to the nucleus to bind DNA at specific DNA motifs and thereby regulate BMP-target genes. Co-regulators might interact with this complex and modulate BMP-mediated gene expression. SMAD6/7 can inhibit the phosphorylation of SMAD1/5/8 and subsequently inhibit BMP pathway.

Upon binding of the ligand, the type II receptor recruits and phosphorylates the type I receptor on specific serine and threonine residues in the intracellular juxtamembrane region. Signalling in most cells by TGF-β occurs through TGF-β type II receptor (TβRII) and TβRI (also termed activin receptor like kinase (ALK)5) complex, activins through activin receptor type IIA (ActRIIA), ActRIIB and ALK4, and BMPs through BMP type II receptor (BMPRII), ActRIIs and ALK1, 2, 3 and 6. However, TGF-β can signal via both ALK1 and ALK5 in ECs (Goumans et al., 2002[33]). Activation of the type I receptor results in recruitment and phosphorylation of receptor-regulated Smads (R-Smads) at the two serine residues located in their extreme carboxyl termini. ALK4, 5 and 7 mediate R-Smad2 and 3 phosphorylation, whereas ALK1, 2, 3 and 6 induce phosphorylation of the R-Smads 1, 5 and 8. Activated R-Smads form complexes with the coSmad i.e. Smad4 which translocate into the nucleus where they act as transcription factors and regulate transcription of target genes. Inhibitory (I) Smads, i.e. Smad6 and -7 are known to compete with R-Smads for type I receptor interaction and mediate proteosomal degradation of type I receptors by recruiting Smurf1/2 E3 ubiquitin ligases, and thereby mitigate activation of the R-Smads (Heldin et al., 1997[38]; Heldin and Moustakas, 2012[39]; Shi and Massagué, 2003[75]; Schmierer and Hill, 2007[72]; Goumans et al., 2009[31]). For a more detailed description of TGF-β signalling, which is not discussed thoroughly here, we refer to recent reviews (Hata Hata and Liebermann, 2015[37]; Euler, 2015[27]; Zhang et al., 2013[93]; Cai et al., 2012[10]). A schematic representation of TGF-β/BMP signalling is depicted in Figure 1(Fig. 1).

### TGF-β signalling in vascular cells

#### Endothelial cells

TGF-β signalling has been implicated in vascular development during embryogenesis, postnatal angiogenesis, and in maintenance of homeostasis during adult life. Mice deficient in TGF-β and TGF-β receptors die during embryogenesis due to defects in yolk sac angiogenesis (Dickson et al., 1995[22]; Oshima et al., 1996[62]; Larsson et al., 2001[50]). Endothelial specific knock out of TβRII or ALK5 mimics the total knock out, demonstrating the importance of TGF-β signalling in endothelial cells (Carvalho et al., 2007[13]). TGF-β modulates proliferation, migration, apoptosis, permeability and morphogenesis of endothelial cells (ECs) (Pardali and ten Dijke, 2012[67]). Increased levels of circulating TGF-β has been found in several CVD, including PAH, HHT, obesity and diabetes (ten Dijke et al., 2008[81]). Interestingly, elevated circulating TGF-β promotes oxidative stress and dysfunction in apolipoprotein E-knockout (ApoE-KO) mice. However, TGF-β can acts either be protective or detrimental in ECs depending on the cell and context (Yan et al., 2014[91]). 

TGF‐β plays a crucial role in the regulation of activation state of ECs through activation of two type I receptors, ALK5 and ALK1. ALK5 is broadly expressed in many tissues, while the expression of ALK1 is largely restricted to the endothelium. It has been demonstrated that stimulation of ALK1 triggers the phosphorylation of Smad1/5/8, whereas ALK5 induces Smad2/3 phosphorylation in ECs (Goumans et al., 2002[33]). ALK1 and ALK5 interact with each other and elicit opposite responses in ECs which eventually results in a fine tune EC function. Of note, mutations in the ALK1 or endoglin are associated with the vascular disorder HHT. In addition to these canonical signalling pathways, TGF‐β may also regulate biological functions in a non-Smad dependent manner such as nicotinamide adenine dinucleotide phosphate (NADPH) oxidase-dependent redox mechanisms (Yan et al., 2014[91]). 

Several lines of evidence show that TGF-β can stimulate or inhibit angiogenesis in vitro and in vivo, depending on the experimental setup. For example, TGF-β/ALK5 signalling inhibits angiogenesis by inhibiting EC proliferation, migration and tube formation in a Smad dependent manner (Goumans et al., 2002[33], 2003[32]). Activation of ALK5 inhibits migration of ECs through enhanced expression of fibronectin and plasminogen activator inhibitor type 1 (PAI-1). ALK5 has also been involved in vascular permeability through modulation of tight junction protein Claudin-5 (Ota et al., 2002[63]). Indeed, ALK5 augments TGF-β mediated vascular permeability and plays critical role in actin cytoskeleton remodelling. Furthermore, TGF-β/ALK5 signalling is also implicated in stabilization of the vessel wall via increased expression of VE-cadherin (Birukova et al., 2005[7]; Rudini et al., 2008[71]). TGF-β induces apoptosis in pulmonary microvascular ECs, which involves the ALK5-Smad2 pathway and decreased expression of the anti-apoptotic genes Bcl-2 and cFLIP. In contrast, several other reports demonstrated the role of the p38 mitogen-activated protein kinase (MAPK) pathway in regulation of TGF-β-induced EC apoptosis (Hyman et al., 2002[40]; Ferrari et al., 2006[28]). These observations demonstrate that TGF-β signalling regulates EC functions in a specific ligand- and context-dependent manner. 

BMPs also play crucial role in the modulation of EC function. Cumulative evidence shows that BMP4, BMP6 and BMP9 are involved in proliferation, migration and angiogenesis of ECs (Valdimarsdottir et al., 2002[82], Suzuki et al., 2008[77]). Inhibition of the BMPRII expression in ECs resulted in reduced phosphorylation of Smad1/5/8 and Id1 expression following hypoxia. Interestingly, similar characteristics were observed in the ECs from PAH. Indeed, mutations in BMPR2 are observed in ~80 % of patients of hereditary PAH (Teichert-Kuliszewska et al., 2006[79]). Therefore, developing new therapeutic strategies that lead to an increase in cell surface BMPRII expression might be beneficial for these patients. The TGF-β co-receptor endoglin regulates the proliferation, migration, apoptosis and angiogenesis of ECs. It has been demonstrated that ECs derived from endoglin deficient embryos have reduced proliferation and migration capacities (Goumans et al., 2009[31]; Yan et al., 2014[91]). Interestingly, the vascular dysfunction in pre-eclampsia is in part caused by the presence of high levels of soluble endoglin (ten Dijke and Arthur, 2007[80]).

#### Smooth muscle cells

Several type I and type II receptors of the TGF-β family are expressed in vascular SMCs (VSMCs). Numerous studies reported that TGF-β/Smad signalling plays key role in differentiation and “phenotype switching” of SMCs via modulation of a large set of SMC differentiation marker genes such as SM-actin, SM22α and calponin (Owens et al., 1995[64]). Disruption of TGF-β signalling leads to defective vessels losing integrity of the vessel wall due to the failure in recruitment and differentiation of SMCs. Genetic deletion of TβRII specifically in VSMCs resulted in vascular defects in the yolk sac; however, these were observed at later stages of development than the total knock out (Carvalho et al., 2007[13]). Whereas mice completely depleted of TβRII die at E10.5, embryos without TβRII in VSMCs are able to survive till E12.5. Besides its role during development, TGF-β is an important regulator of the differentiation status of SMCs in adult animals. For detailed description of the effect of TGF-β signalling on SMC differentiation we like to refer to Guo and Chen (2012[34]). 

Myocardin, a coactivator of serum response factor and δEF, a zinc finger E-box binding transcription factor, have been shown to increase TGF-β/Smad-3 induced activation of SM22α expression (Qiu et al., 2005[70]; Nishimura et al., 2006[61]). TGF-β also blocks proliferation of VSMCs via ALK5/Smad3/p38 MAPK pathway. However, TGF-β inhibits migration of SMCs in a Smad3-independent manner through enhanced expression of cysteine rich protein 2 (Lin et al., 2008[56]). Interestingly, SMCs derived from Smad3-deficient mice show reduced inhibition of proliferation, but no effect on migration following treatment with TGF-β (Kobayashi et al., 2005[46]). Recent studies reported that activation of TGF-β/Smad3 pathway inhibit VSMC apoptosis through an autocrine signalling mechanism involving VEGF-A following angioplasty (Shi et al., 2014[74]). 

Endoglin and BMPs also play an important role in the formation, differentiation and function of VSMCs. The BMP signalling pathway is also implicated in the induction and maintenance of the contractile SMCs and loss of BMP signalling is associated with abnormalities in vascular development and in vascular proliferative conditions, such as restenosis and PAH (International PPH Consortium et al., 2000[42]; Koehler et al., 2004[47]). The effect BMP has on VSMCs is context dependent. BMP2 and BMP7 can inhibit proliferation of VSMCs whereas BMP2 promotes migration of VSMCs. However, BMP4 and BMP7 trigger apoptosis via a caspase 8/9-dependent mechanism in pulmonary SMCs, demonstrating that how BMPs effect the function of SMCs is depending on the source of SMCs and their local environment (Lagna et al., 2006[49]; Goumans et al., 2009[31]; Cai et al., 2012[10]). 

## TGF-β regulation of miRNAs in vascular cells

While miRNAs regulate the expression of several members of the TGF-ί signalling pathway, TGF-ί itself can modulate the expression of numerous miRNAs in multiple cell types. TGF-ί regulates their expression by binding to p68, a component of the Drosha microprocessor complex (Butz et al., 2012[9]; Davis et al., 2008[18]). MiRNAs that are regulated by TGF-ί in vascular cells are summarized in Table 1(Tab. 1) (References in Table 1: let-7d: Pandit et al., 2010[65]; miR-21: Kumarswamy et al., 2012[48]; miR-24: Wang et al., 2012[84]; miR-27b: Wang et al., 2012[85]; miR-29a; Wang et al., 2013[84]; miR-143/145: Long and Miano, 2011[57]).

## Regulation of members of the TGF-β superfamily by miRNAs in vascular cells

Although several predictions were made for potential miRNAs that target members of TGF-β family based on *in silico* analysis, experimental validation is crucial. Numerous recent studies show that several miRNAs target components of TGF-β signalling pathway in multiple cells including ECs and SMCs, and play a pivotal role in the pathogenesis of a variety of vascular diseases. miRNAs targeting TGF-β signalling pathway in vascular cells and associated vascular diseases are summarized in Table 2(Tab. 2)(References in Table 2: ECs: Liao et al., 2014[55]; Chen et al., 2012[16]; Brock et al., 2009[8]; Di Bernardini et al., 2014[21]; Drake et al., 2013[26]; Tabruyn et al., 2013[78]; Icli et al., 2013[41]; SMCs: Pullamsetti et al., 2011[69]; Kang et al., 2012[43]; Ahmed et al., 2011[1]; Yang et al., 2012[92]; Caruso et al., 2010[12]; Chan et al., 2010[14]; Leeper et al., 2011[51]; Merk et al., 2012[58]; Balderman et al., 2012[5]; Kim et al., 2014[45]; Nishiguchi et al., 2015[60]; Wang et al., 2013[87]; Davis-Dusenbery et al., 2011[19]; Caruso et al., 2012[11]; Kang et al., 2012[44]; Other vascular cells: Bai et al., 2013[4]; Liang et al., 2012[54]; Shan et al., 2009[73]; Zheng et al., 2010[94]; Li et al., 2013[53]; Shan et al., 2009[73]).

### Endothelial cell miRNAs

Endothelial cells are crucial for maintaining vascular homeostasis. miRNAs are expressed in the vasculature and are indispensable for endothelial regulation of vessel function through regulation of key pathways, including TGF-β/BMP signalling. We will now describe the most important miRNAs in more detail.

### Let-7g

Members of the Let-7 family are highly-conserved miRNAs and play crucial role in regulation of cell differentiation (Liao et al., 2014[55]). Although several studies implicate a role for the Let-7 family in many pathologies, their role in CVD remains unknown. Recently, Liao et al. (2014[55]) demonstrated the involvement of Let-7g, a well-studied members of the Let-7 group, in ECs. Let-7g has been shown to effect on multiple EC functions through targeting three key components of TGF-β pathway. Overexpression of Let-7g down-regulates the expression of thrombospondin 1(THBS1), Tgfbr1, and Smad2 genes in ECs. TGF-β1 induced phosphorylation of Smad2 (pSmad2) is decreased upon overexpression of Let-7g in ECs. Conversely, inhibition of Let-7g enhanced TGF-β1 induced pSmad2, further suggesting that Let-7g is a key player in the modulation of TGF-β1 signalling in ECs. Ectopic expression of Let-7g significantly decreased vascular cell adhesion molecule-1 (VCAM-1) secretion, thereby inhibiting the monocyte adhesion to ECs. Moreover, Let-7g markedly attenuated inflammation and enhanced angiogenesis in a TGF-β dependent manner. Let-7g also reduces the expression of plasminogen activator inhibitor (PAI)-1 in ECs, an important downstream effector of the TGF-β pathway. Injection of Let-7g-expressing plasmids into ApoE-KO mice revealed that the protein levels of PAI-1 and pSmad2 are decreased in the vessel wall. In contrast, inhibition of Let-7g into ApoE-KO mice resulted in excessive growth of vascular intima-media, enhanced infiltration of macrophages, and induction of TGF-β downstream genes such as PAI-1 in the carotid arteries. In line with these observations, serum levels of Let-7g are inversely correlated with PAI-1 levels in lacunar stroke patients, who have EC dysfunction. Interestingly, Let-7g promotes senescence of ECs through augmenting sirtuin-1 (SIRT-1) protein levels. Based on these observations, Liao et al. (2014[55]) proposed that Let-7g might be a potential target in CVD through its protective function in ECs via modulation of TGF-β signalling.

Another study reported that fibroblast growth factor (FGF) signalling controls TGF-β signalling via regulation of let-7 expression in ECs (Chen et al., 2012[16]). Basal FGF signalling is essential for maintaining the expression of let-7 in ECs. Disruption of the intracellular adaptor FGF receptor substrate 2 (FRS2) of FGF signalling results in enhanced the expression of TβR1 and induces pSmad2. Defective FGF signalling also induces the expression of let-7, which in turn inhibits TGF-β signalling and suppresses several markers of endothelial-to-mesenchymal transition (Endo-MT), a process in which endothelial cells lose their cobble stone morphology and start to express SMC markers. Endo-MT is pivotal process involved in the formation of neointima formation, which underlies in several diseases such as PAH, transplant vasculopathy, restenosis, and atherosclerosis among others. Therefore, regulation of the FGF and TGF-β axis via let-7 may play a crucial role in vessel wall homeostasis (Chen et al., 2012[16]). 

### miR-17/92

PAH is a fatal disorder of the lung vasculature characterized by enhanced proliferation of ECs and SMCs. Mutations in the BMPR2 gene have been identified in about 80 % of hereditary PAH and 10-40 % of sporadic idiopathic PAH patients (International PPH Consortium et al., 2000[42]; Koehler et al., 2004[47]). The miRNA cluster 17/92 (miR-17/92), containing six mature miRs: miR-17, miR-18a, miR-19a, miR-19b-1, miR-20a, and miR-92, has been shown to target the BMPR2 gene and reduces protein expression of BMPR2 as well as 3′-UTR activity of BMPR2 (Brock et al., 2009[8]). Interestingly, the pro-inflammatory cytokine interleukin (IL)-6 induces the expression of miR-17/92 cluster genes in human pulmonary artery ECs (HPAECs). Knockdown of signal transducer and activator of transcription (STAT)-3, a potential modulator of IL-6 signalling, resulted in inhibition of the IL-6 induced expression of miR-17/92. In agreement with this, a highly conserved STAT3-binding site was found in the promoter region of the miR-17/92 gene C13orf25. IL-6 significantly increased the expression of C13orf25 through this distinct region. Furthermore, it was shown that STAT3 activation results in reduced protein expression of BMPR2. These data indicate that the STAT3-miR-17/92 axis regulates BMPR2 expression in HPAECs and therefore may be useful as a target for designing therapeutic strategies for PAH to increase the reduced expression levels of BMPRII (Brock et al., 2009[8]).

### miR-21

Cardiovascular regeneration is an essential repair or replacement process that involves the regeneration of damaged vessels and formation of new vessels in the infarcted area. However, identification of a suitable EC cell source for cardiovascular regeneration has proven to be extremely challenging. Recently, Di Bernardini et al. (2014[21]) reported that miR-21 regulates differentiation of inducible pluripotent stem cells (iPSCs) to ECs through directly targeting TGF-β2. TGF-β2 has been shown to be involved in inducing several EC markers and in tube formation in vitro in iPSCs. Overexpression of miR-21 in pre-differentiated iPSCs up-regulates the expression of several EC markers, whereas inhibition of miR-21 exerts opposite effects. Ectopic expression of miR-21 enhanced TGF-β2 expression. Knockdown of TGF-β2 attenuated miR-21 induced EC marker expression in iPSCs. These findings suggest that miR-21 in concert with TGF-β2 regulates the differentiation of iPSCs into ECs (Di Bernardini et al., 2014[21]). 

In addition to its important role in iPSCs differentiation, miR-21 is also implicated in pulmonary hypertension (PH) by regulating the BMP pathway in ECs (Parikh et al., 2012[68]). Parikh et al. (2012[68]) identified miR-21 as a potential player in PH using multiple approaches including network analysis, rodent models and patient samples. In HPAECs, BMPR2 signalling induces the expression of miR-21 which in turn suppresses expression of BMPR2. In particular, BMP9 induces the expression of miR-21 and knockdown of BMPR2 significantly mitigates miR-21 expression in HPAECs. In addition, knockdown of Smad5, a downstream effector of BMPRII signalling also decreases expression of miR-21. Interestingly, Smad4 depletion did not influence miR-21 expression, suggesting that BMPR2 is required for inducing the expression of miR-21 upon BMP stimulation, and this process depends on certain, but not all, Smads (Parikh et al., 2012[68]).

### miR-27 and miR-205

miR-27 has been shown to be expressed in vascular cells including ECs. In pulmonary ECs, BMP9 strongly induces the expression of miR-27a (Drake et al., 2013[26]). As PAH is associated with BMPR2 mutations accompanied by enhanced proliferation of ECs and SMCs, Drake et al. (2013[26]) explored the effect of miR-27 on distinct PAH mutations. They reported that miR-27a expression is decreased in ECs of PAH patients with different mutations of the BMPR2. However, treatment with ataluren, an investigational drug, significantly enhances miR-27 processing across a range of nonsense PAH mutations in a BMP dependent manner. Ataluren has been shown to induce read through of nonsense mutations by altering ribosomal proofreading activity only on premature termination codons, but does not affect bona fide termination codons. Ataluren treatment enhances the protein levels of BMPR-II and subsequently phosphorylation of Smads. This results in correction of the BMP-mediated miR processing and inhibits the excessive proliferation of pulmonary ECs and SMCs derived from PAH patients with nonsense mutations. These data identified ataluren as a capable drug to treat PAH as it can effectively suppresses BMPR2 and SMAD9 nonsense mutations and correct several aspects of BMP signalling through regulation of miR-27 in ECs (Drake et al., 2013[26]).

In addition to its role in PAH, miR-27 together with miR-205 has been implicated in HHT, which is an autosomal dominant vascular disorder that leads to formation of abnormal blood vessel formation and haemorrhages (Tabruyn et al., 2013[78]; Dingenouts et al., 2015[23]). When analysing the plasma of HHT1 and HHT2 patients, the expression level of miR-27a was higher whereas miR-205 was lower compared to controls (Tabruyn et al., 2013[78]). Smad1 and Smad4 have been identified as targets of miR-205. Indeed, ectopic expression of miR-205 reduces protein expression of Smad1 and Smad4, but not their mRNA levels. Conversely, inhibition of miR-205 strongly increases protein levels of Smad1 and Smad4, but mRNA levels were not affected. Overexpression of miR-205 attenuates proliferation, migration and tube formation of ECs. These findings suggest that both miR-27a and miR-205 might be useful as biomarkers as well as targets for treatment of HHT patients (Tabruyn et al., 2013[78]).

### miR-26a

The expression of miR-26a is induced in a murine model of acute myocardial infarction and also in patients with acute coronary syndromes (Icli et al., 2013[41]). miR-26a specifically targets Smad1, but not other Smad family members, including Smad2, Smad4, and Smad7. Ectopic expression of miR-26a decreased the 3′-UTR activity of Smad1 which results in reduced expression of Id1 and enhanced expression of p21WAF/CIP and p27. Overexpression of miR-26a in ECs significantly enhanced the proliferation, but inhibited migration, tube formation and angiogenesis. In contrast, the inhibitor of miR-26a displayed opposite effects. These in vitro results were further substantiated by in vivo findings in zebrafish and mice. In zebrafish, ectopic expression of miR-26a resulted in reduced formation of the caudal vein plexus, a BMP driven process, which was rescued by Smad1 overexpression. In mice, overexpression of miR-26a decreased expression of Smad1 in ECs as well as exercise-induced angiogenesis. Conversely, inhibition of miR-26a enhanced expression of Smad1 and promotes robust angiogenesis, which is associated with decreased myocardial infarct size and improved heart function, suggesting that miR-26a acts as a critical regulator of angiogenesis in ECs in a Smad1 dependent manner (Icli et al., 2013[41]).

### Smooth muscle cell miRNAs

As described above, TGF-β/BMP signalling is implicated in the differentiation of SMCs into contractile SMCs. Many miRs are involved in the phenotypic switch of SMCs in diverse vascular pathologies such as restenosis, atherosclerosis, and PAH through regulation of TGF-β/BMP signalling. The functional expression of miRs has been associated with development of SMCs. Indeed, a mounting body of evidence shows that lack of the miR processing enzyme 'Dicer' in SMCs during development results in late embryonic lethality and haemorrhage, probably due to reduced proliferation and differentiation of SMCs (Albinsson et al., 2010[3]). We will discuss several microRNAs involved in SMC function in relation to TGF-β signalling in more detail.

### miR-17/92

Although the miR-17/92 cluster has been implicated in ECs, recent reports suggest that this cluster is also involved in pulmonary artery SMCs (PASMCs) related to the pathology of PAH (Pullamsetti et al., 2012[69]). Pullamsetti and colleagues (2012[69]) studied the role of miR-17 in PAH using antagomiRs in both mouse and rat models of PAH. Although the treatment with antagomiRs of miR-17 enhanced expression of BMPR2, it did not effect TGFβR2 mRNA levels. Smad5 was also predicted to be a target of miR-17 by *in silico* analysis, however treating animals with a miR-17 inhibitor did not effect Smad5 mRNA levels. Overall, these findings suggest that miR-17 regulates many genes, most likely influences different cell types of the lung and therefore miR-17 might serve as an effective target for the treatment of PH. 

### miR-21

Vascular proliferative disorders such as restenosis and vein graft disease are associated with excessive proliferation and migration of SMCs. There is compelling evidence that miR-21 is involved in modulation of SMC function. BMP-4 has been shown to induce the expression of miR-21 which in turn triggers the expression of several SMC contractile genes by targeting programmed cell death 4 (PDCD4) (Kang et al., 2012[43]). It was also reported that miR-21 targets many members of the dedicator of cytokinesis (DOCK) superfamily. Overexpression of miR-21 decreases the expression of Dock-4, 5 and 7 and thereby reduces the migration of cells through repressing the activity of GTPase Rac1, a known player in cytoskeletal organization and cell migration. Interestingly, miR-21 modulates the contractile function of SMCs by targeting Dock-4 and 5 through a Rac1-independent mechanism. Of note, miR-21 prevented the inhibitory effects of BMP4 on proliferation and migration of primary keratinocytes and HaCaT cells (Ahmed et al., 2011[1]). This observed discrepancy could be attributable to different cell types involved in distinct pathologies and requires more research. Taken together these data suggest that miR-21 plays a key role in the phenotypic switch of SMCs through DOCK family proteins in a BMP dependent manner (Kang et al., 2012[43]).

The study from Kang et al. (2012[43]) is further corroborated by a recent study on the role of miR-21 in SMC function. Stein et al. (2014[76]) demonstrated that miR-21 plays a pivotal role in the thrombospondin-1 (TSP-1) induced proliferation and migration of VSMCs. However, the activity of miR-21 is not essential for TSP-1 to induce the expression of prostenotic genes including TGF-β2 (Stein et al., 2014[76]). Further studies are warranted to understand these findings. 

Although it has been reported that miR-21 modulates PH through its effects on BMP signalling in HPAECs, another study showed that miR-21 also plays an essential role in PASMCs via BMP signalling, and thereby modulates hypoxia induced PH (Yang et al., 2012[92]). Yang et al. (2012[92]) demonstrated that miR-21 expression is up-regulated in distal small arteries in the lungs of hypoxia-exposed mice. Moreover, there is a reciprocal relationship between BMPR2 and miR-21 expression, further supporting the previous observations. Furthermore, it was also shown that miR-21 induces proliferation of PASMCs, mostly like through modulation of BMP signalling. However, further research is necessary to validate the role of miR-21 in PASMCs. 

### miR-22

TGFBR1 has been identified as a target for miR-22, which is down-regulated after 21 days in a rat hypoxic model of PAH and after 2 days in a monocrotaline injection model of PAH. Interestingly, TGF-β1 stimulation of PASMCs induced down-regulation of miR-22 similar to hypoxic and monocrotaline-treated rats. More studies are required to investigate the role of miR-22 on TGFBR1 in PAH and also the effect of TGF-β1 on miR-22 expression (Caruso et al., 2010[12]). 

### miR-24

PDGF-BB induces the expression of miR-24 in vascular SMCs (VSMCs) which subsequently reduces the expression of Tribbles-like protein-3 (Trb3). Inhibition of Trb3 results in decreased TGF-β and BMP signalling through reduced expression of Smad proteins and thereby induce synthetic phenotype in VSMCs (Chan et al., 2010[14]). Inhibition of miR-24 induces Trb3 expression and pro-synthetic activity of PDGF signalling. In contrast, overexpression of miR-24 inhibits Trb3 in BMP-independent manner and represses pSmads in BMP-dependent manner resulting in inhibition of the BMP-Smad pathway in PASMCs. Overexpression of miR-24 suppressed BMP4-induced SMA and Id3 expression. Ectopic expression of Trb3 decreased the inhibitory effect of miR-24 on the BMP4-mediated induction of SMA and Id3, suggesting a potential role of the miR-24-Trb3 axis in the regulation of the BMP pathway. Furthermore, miR-24 inhibits BMP4-mediated proliferation and BMP-4 induced actin remodelling of PASMCs, indicating a role of miR-24 in different pro-contractile activities of the BMP4 pathway in VSMCs. Interestingly, miR-24 negatively regulates the expression of total Smad2, Smad3, and pSmad2, and thereby inhibits the TGF-β-signalling pathway. miR-24 also inhibits the TGF-β-induced expression of miR-21. Furthermore, PDGF-BB modulates the TGF-β-mediated pro-contractile function via regulation of Trb3 and Smads via up-regulation of miR-24 (Chan et al., 2010[14]). 

### miR-26

In addition to its potential role in ECs, miR-26a also plays crucial role in the 'phenotypic switch' of SMCs which plays a key role in the formation of abdominal aortic aneurysm (AAA) (Leeper et al., 2011[51]). Ectopic expression of miR-26a abolished the differentiation of SMCs whereas inhibition of miR-26a promoted differentiation and apoptosis accompanied by reduced proliferation and migration of SMCs. Overexpression of miR-26a reduced whereas inhibition of miR-26a enhanced Smad signalling. Specifically, inhibition of miR-26a enhanced expression of Smad1 and Smad4 whereas overexpression of miR-26a abolished Smad1 expression. The expression of miR-26a is reduced in the aneurysm wall as tested in two mouse models of AAA, associated with a phenotypic switch of SMCs. miR-26a enhances proliferation and inhibits differentiation and apoptosis of SMCs, and modulates TGF-β pathway. These findings suggest that miR-26a plays a crucial role in SMC biology and might represent a potential therapeutic target in AAA disease (Leeper et al., 2011[51]).

### miR-29b 

miR-29b is also implicated in the development of aneurysms. It has been demonstrated that miR-29b expression is induced in the ascending aorta of “Marfan” mice (Fbn1C1039G/+) (Merk et al., 2012[58]). Ascending aortas of Marfan mice displayed reduced and fragmented elastin protein levels and decreased mRNA levels of elastin and enhanced matrix metalloproteinase-2 (MMP-2) expression and activity. Both elastin and matrix metallo-protease MMP-2 are identified as targets of miR-29b. Interestingly, NFκB, a pivotal inflammatory transcription factor acts as a repressor of miR-29b. TGF-β treatment inhibits NFκB and thereby enhances miR-29b expression aortic SMCs derived from Marfan mice. In line with these results, treatment with an NFκB inhibitor augmented, whereas blocking TGF-β or losartan treatment strongly decreased the expression of miR-29b in Marfan mice. Inhibition of miR-29b resulted in reduced development of aneurysms, aortic wall apoptosis, and extracellular matrix deficiencies. Taken together, this study identified miR-29b as a novel target for development of therapeutic strategies for aneurysms (Merk et al., 2012[58]). 

### miR-30

Dedifferentiation of SMCs to an osteoblast-like phenotype may result in vascular calcification under some pathological conditions. A miR microarray analysis identified miR-30b and miR-30c as miRs that regulate expression of Runx2, a key transcription factor involved in calcification. Treatment of SMCs with BMP-2 attenuates expression of miR-30b and miR-30c in SMCs (Balderman et al., 2012[5]). In addition, it has been reported that miR-30b and miR-30c binds to the 3′-UTR of Runx2. Consistent with this notion, antagomiRs targeting miR-30b and miR-30c significantly increased the expression of Runx2 and results in intracellular calcium deposition and mineralization. Moreover, ectopic expression of miR-30b and miR-30c reduced the expression of Runx2 and mineralization of SMCs. To substantiate these in vitro findings and to overrule the tissue cell culture effects, immune-histochemical stainings were performed for expression of BMP-2 and miR-30b in calcified human coronary arteries. They found that BMP-2 is highly expressed whereas miR-30b is lowly expressed in calcified human coronary arteries. This study supports the notion that BMP-2 reduces the expression of miR-30b and miR-30c to enhance expression of Runx2 in SMCs and thereby promote mineralization (Balderman et al., 2012[5]).

### miR-96

Another miR involved in phenotypic switch of SMCs is miR-96. BMP4 down-regulates the expression of miR-96 in VSMCs. miR-96 targets Trb3 gene which regulates BMP signalling pathway. miR-96 has been shown to induce contractile phenotype in VSMCs through regulation of SMC‐specific genes. Because phenotype switching of SMCs is crucial in diseases such as atherosclerosis and restenosis, miR-96 might play a role in these diseases (Kim et al., 2014[45]). Indeed, several recent studies suggest that miR-96 is involved in platelet reactivity, aggregation, secretion and adhesion and thereby plays essential role in the arterial thrombotic conditions such as myocardial infarction (Nishiguchi et al., 2015[60]). Another study demonstrated the indirect involvement of miR-96 in vascular diseases through modulation of cholesterol metabolism via inhibition of selective high-density lipoprotein cholesterol (HDL-C) uptake and SR-BI expression in human hepatic cells (Wang et al., 2013[87]). However, the role of BMP signalling in the miR-96-mediated effects was not explored. 

### miR-143/145

Kruppel-like factor-4 (KLF4) has been implicated in the phenotypic switch of SMCs through modulation of SMC contractile genes. Both TGF-β and BMP4 strongly reduces the expression of KLF4 through inducing the expression of miR-143/145 in VSMCs (Davis-Dusenbery et al., 2011[19]). Inhibition of miR-145 by antagomiRs enhanced the expression of KLF4, which in turn increases expression of SMC contractile genes. Consistent with previous reports, these data suggests that KLF4 is pivotal for the TGF-β and BMP4-mediated phenotypic switch of SMCs. Although TGF-β and BMP4 enhance the expression of miR-143/145 through the CArG box, TGF-β mediates its effects through up-regulation of myocardin whereas BMP4 triggers nuclear translocation of MRTF-A, indicating the potential similarities and the differences of these pathways in modulation of KLF4-mediated phenotypic switch of SMCs (Davis-Dusenbery et al., 2011[19]).

Another report demonstrated that miR-145 is expressed in SMCs of mouse lungs. Hypoxia, one of the triggers of PAH, also induces miR-145 expression in mouse lungs. Mice deficient for miR-145 and mice treated with inhibitors of miR-145 are resistant to the development of PAH. PASMCs derived from PAH patients with BMPR2 mutation showed enhanced expression of miR-145, suggesting that there is an inverse correlation between miR-145 expression and BMPR2 levels. Interestingly, inhibitors of miR-143 had no effect on PAH development (Caruso et al., 2012[11]). 

### miR-302

The miR-302 family has been implicated in PAH. BMP4 decreases the expression of miR-302 in a Smad-dependent manner. Members of the miR-302 family have been shown to target BMPR2, which results in reduction in transcription of BMPR2 that leads to decreased BMP signalling in these cells. Overexpression of miR-302 decreases BMP4-mediated cell proliferation and migration which are two key characteristic features in PAH, suggesting that miR-302 might be beneficial in the treatment of PAH and other vascular proliferative diseases (Kang et al., 2012[44]). 

## Other vascular cells

### miR-17/92

VEGF has been shown to be a direct target of the miR-17/92 cluster which is regulated by BMP signalling in the second heart field (SHF) that plays essential role in the outflow tract. Defects in outflow tract leads to congenital heart disease. Bai et al. (2013[4]) also reported that deletion of BMP4 and BMP7 is associated with defective epithelial to mesenchymal transition (EMT) in which VEGF is a downstream effector of the BMP pathway. Overexpression of miR-17/92 can partially suppress EMT through regulation of VEGF. These data indicates that the miR-17/92 cluster plays a crucial role in the outflow tract through regulation of BMP pathway and might be beneficial in the treatment of congenital heart disease.

### miR-155

Numerous previous reports suggest that TGF-β and the angiotensin II type 1 receptor (AT1R) are associated in many pathologies including aneurysms and hypertension (Wolf, 1998[89]; Gallo et al., 2014[30]). AT1R has been identified as a miR-155 target gene (Zheng et al., 2010[94]). miR-155 modulates expression of AT1R and plays a role in phenotypic differentiation of rat aortic adventitial fibroblasts (AFs). Ectopic expression of miR-155 inhibits AT1R 3′-UTR activity reporter activity and reduces protein levels of AT1R, but not mRNA levels. Although, overexpression of miR-155 inhibits Ang II-induced αSMA expression, it did not influence TGF-β induced αSMA expression (Zheng et al., 2010[94]). 

### miR-181c

Expression of miR-181c is induced in human cardiac samples from individuals with ventricular septal defects (VSDs), which leads to congenital heart defects (CHDs). Patients with VSDs show reduced expression levels of BMPR2, which is a predicted target of miR-181c. Indeed, overexpression of miR-181c down-regulated the expression of BMPR2. Because 80 % of hereditary PAH patients show mutations in BMPR2 accompanied by reduced levels of BMPR2 expression, it is helpful to investigate the role of miR-181c on BMPR2 expression for the treatment of PAH (Li et al, 2013[53]). 

## Conlusions and Perspectives

The TGF-β/BMP signalling plays crucial role in a wide-array of biological process, including cell proliferation, migration and differentiation. The discovery and functional characterization of miRs have led us to understand the cellular biology and their effect on diverse signalling pathways supports a role as pivotal regulators of cell function. There is compelling evidence shows that miRs are involved in pathogenesis of CVD and modulation of miRs has shown potential therapeutic benefit in distinct vascular diseases. Both TGF-β and BMP signals modulate the expression of miRs. Conversely, miRs also regulate TGF-β/BMP signalling in vascular cells. The discovery of interaction between the TGF-β/BMP signalling and miRs in CVD is an exciting area of research. Understanding the underlying mechanism how TGF-β/BMP signalling modulates miRs and vice versa might be crucial for normal development, maintaining homeostasis, and treatment for vascular pathologies. 

In the current review, we summarized the miRs regulation of TGF-β/BMP signalling in vascular cells including ECs and SMCs, two key cell types involved in many vascular diseases. Many miRs target distinct components of the TGF-β/BMP signalling such as Smad1, Smad4, Smad5, and BMPR2. This extensive information have led us to understand the underlying mechanism through which miRs are integrated into the TGF-β/BMP signalling. However, the functional roles of TGF-β/BMP signalling associated miRs in vascular cells still need to be explored in detail using in vivo models and patient samples. Although many miRs are associated with TGF-β/BMP signalling, there is still long way to go. Future studies are required to explore the interactions and precise mechanisms of the TGF-β/BMP signalling and miRs in CVD. Understanding this may help in the development of miRNA-based novel therapeutic approach through modulating the TGF-β/BMP signalling for patients with CVD.

Cellular composition of the cardiovascular system changes under the diseased conditions partly through modulation of TGF-β/BMP signalling and results in differential expression patterns of miRs. Although the mechanisms modulating cellular composition and release of miRs are poorly understood, circulating miRs are stable and may reflect the initiation and progression of the disease and could therefore useful as biomarker to aid in diagnosis and monitoring disease progression and response to treatment. Some of the major limitations in miR research is that lack of specificity and unpredictable levels of miRs in vivo. These potential barriers must be overcome before their full therapeutic potential may be realized. Recent reports suggest that clinical application of miRs is promising including the use of biomarkers and clinical trials with miR inhibitors. Although extensive in vitro data implicate a role of miRs in CVD, testing the miRs in vivo in disease models is essential. In conclusion, the involvement of miRs in regulation of TGF-β/BMP signalling in CVD warrant further genetic and pharmacological elucidation of their function in vivo in order to develop miRNA-based approaches for the prevention and treatment of cardiovascular disease.

## Acknowledgements

Research on the role of TGF-β family members in cardiovascular diseases in our laboratory is supported by the Netherlands CardioVascular Research Initiative: the Dutch Heart Foundation, Dutch Federation of University Medical Centers, the Netherlands Organization for Health Research and Development (PHAEDRA grant). We thank Mr. Marcus Kenyon for help with schematic illustration.

## Conflict of interest

The authors declare that they have no conflict of interest.

## Figures and Tables

**Table 1:**
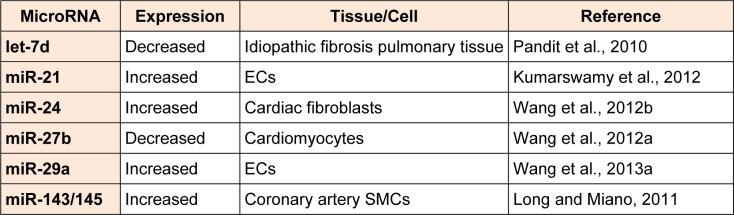
miRNAs that are regulated by TGF-β/BMP signalling pathway in vascular cells

**Table 2:**
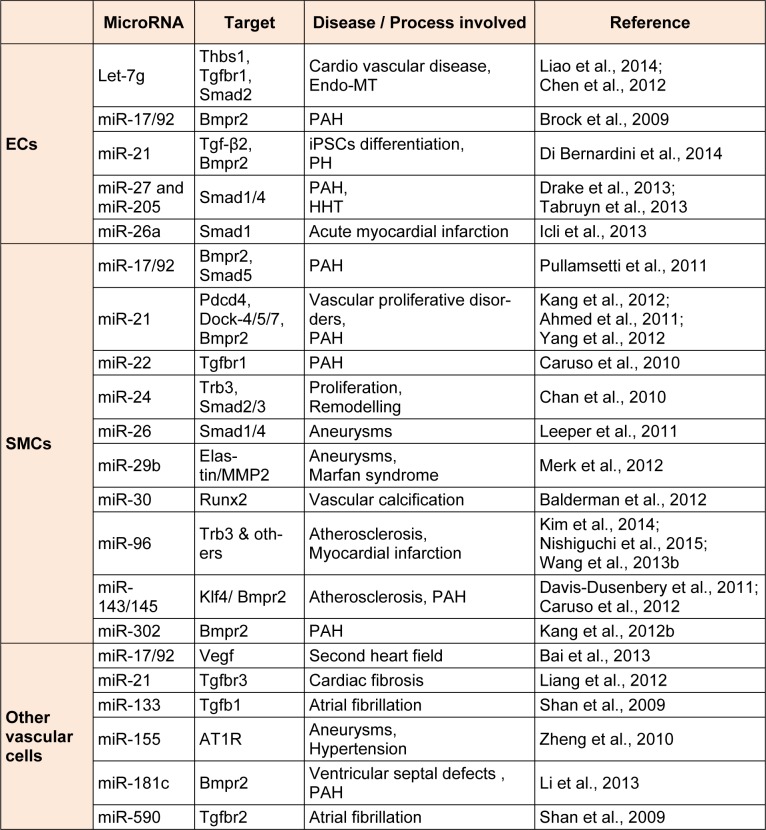
miRNAs targeting TGF-β/BMP signalling pathway in vascular cells
